# Dynamic changes of viral load and the duration of viral shedding in patients with hand, foot and mouth disease: a protocol for longitudinal study

**DOI:** 10.1186/s12879-022-07131-w

**Published:** 2022-02-20

**Authors:** Xiaoxia Duan, Chaoyong Zhang, Zhenhua Chen, Juan Liao, Yilan Zeng, Weiwei Huang, Xueling Ren, Xueqin Tang, Hongxia Peng, Delan Zhang, Xiao Wang, Ping Yuan, Lu Long

**Affiliations:** 1 Department of Epidemiology and Health Statistics, West China School of Public Health and West China Fourth Hospital, Sichuan University, No.16, Section 3, Renmin south road, Wuhou District, Chengdu, Sichuan China; 2 Public Health Clinical Center of Chengdu, Chengdu, Sichuan China; 3 Department of Microbiology Laboratory, Chengdu Municipal Center for Disease Control and Prevention, Chengdu, Sichuan China; 4 Department of Gastroenterology, West China School of Public Health and West China Forth Hospital, Sichuan University, Chengdu, Sichuan China; 5 Non-Communicable Diseases Research Center, West China-PUMC C.C. Chen Institute of Health, Sichuan University, Chengdu, Sichuan China

**Keywords:** Hand foot and mouth disease, Enterovirus, Viral shedding, Viral load, Bayesian multilevel model

## Abstract

**Background:**

The duration of virus shedding is necessary for determining the infectious period. But there were few quantitative studies on the changes of viral load and the law of the viral shedding in hand foot and mouth disease (HFMD) patients has not yet been clarified.

**Methods:**

This study will prospectively recruit coxsackievirus A10 (CV-A10), coxsackievirus A16 (CV-A16) and coxsackievirus A6 (CV-A6) infected inpatients from January 2022 to December 2022. A series of samples and questionnaire information will be collected regularly to establish the dynamic function relationship between time and viral load changes and a Bayesian multilevel model will be constructed to clarify the evolvement rules which reflect the dynamic changes of viral load and the duration of viral shedding in patients with HFMD.

**Discussion:**

The results of this study is expected to further clarify the evolvement rules which reflect the dynamic changes of viral load and the duration of viral shedding in HFMD patients under the influence of related factors. It can also provide important evidence for the scientific definition of the infectious period and isolation period of HFMD in China.

## Background

Hand, foot and mouth disease (HFMD) is an acute infectious disease caused by a group of enteroviruses. Enterovirus A71 (EV-A71) and coxsackievirus A16 (CV-A16) were the most common viral types in the past few decades [1]. After inactivated EV-A71 vaccines gradually expanded, CV-A16, coxsackievirus A6 (CV-A6) and coxsackievirus A10 (CV-A10) gradually becoming the predominant and co-circulated serotypes [2–4], and the major causative agents of outbreaks and epidemics around the world of HFMD [5–7]. Recently, the incidence of HFMD has declined, but it still ranked second in the number of in Category C infectious diseases in China [8].

No established treatment is available for HFMD [9], as the highly contagious HFMD spreads rapidly among children and always spreads in kindergartens, schools and other collective units, and isolation is the most effective way to control its spread. Therefore, most countries in the Asia–Pacific region, have adopted social distancing measures, such as closures of daycare centers and schools [10]. However, the isolation regulations for the source of infection vary greatly in different countries and regions, and there is no clear definition of the period of infection [10, 11]. The infectious period mainly determined by the duration of virus shedding is an important basis for determining the isolation period of patients with HFMD.

Several small-scale studies have qualitatively explored the duration of viral shedding for HFMD patients induced by EV-A71 and CV-A16, but the results were quite different [12, 13]. In addition, there were few studies that focused on CV-A6 and CV-A10. A previous meta-analysis has showed that most cured HFMD cases continued to excrete the virus for a long time, and there were large differences between individuals [14]. The current isolation period may be extremely limited in blocking the transmission of HFMD [12, 15], and the arbitrary extension of the HFMD isolation period is not only unscientific but will cause significant social impact [16].

This study will prospectively recruit CV-A10, CV-A16 and CV-A6 infected inpatients as the research subjects. A series of samples and questionnaire information will be collected regularly to establish the dynamic function relationship between time and viral load changes. Bayesian multilevel model will be used to clarify the evolvement rules which reflect the dynamic changes of viral load and the duration of viral shedding in HFMD patients under the influence of related factors.

## Aims and hypotheses

We aim to perform a prospective study base on patients hospitalized in the public clinical center of Chengdu to collect a series of samples from patients with HFMD induced by CV-A10, CV-A16 and CV-A6 and evaluate the dynamic changes of viral load and the duration of viral shedding in HFMD patients under the influence of related factors. The results of the study can further provide important evidence for the scientific definition of the infectious period and isolation period of HFMD in China.

## Methods

### Study design

This is a hospital-based prospective cohort study, and study methodological design will follow the requirements of ‘Strengthening the Reporting of Observational Studies in Epidemiology’ (STROBE) checklist for cohort studies [17]. During February 1 to December 31, 2022, we will seek to enroll and trace hospitalized HFMD patients in a designated referral hospital. The demographic and clinical data of HFMD patients during the whole clinical course and series clinical specimens will be collected to evaluate the dynamic changes of viral load and the duration of viral shedding.

#### Study site

The study will be conducted at the public health clinical center of Chengdu, capital city of Sichuan Province in Western China. It is a designated hospital for the diagnosis and treatment of HFMD in Sichuan Province, as well as a referral hospital for critically ill cases. In addition to general enterovirus testing, all hospitalized patients tested for EV-A71, CV-A16, CV-A6 and CV-A10 serotypes to confirm diagnosis since 2018. There were on average 1500 hospitalized HFMD cases annually before 2019, which provides sample size guarantee for the smooth development of the project.

#### Study subjects

All hospitalized patients meeting the following inclusion and exclusion criteria during February 1 to December 31, 2022 will be recruited in this study. The initial minimum sample size of this study is expected to reach about 120–160 cases. If the sample does not meet the minimum required follow-up volume, we will appropriately extend the project time.

##### Inclusion criteria

1. Admitted to public clinical center of Chengdu due to HFMD induced by CV-A6, CV-A10 and CV-A16.
2. Written informed consent from a parent or legal guardian for/and children.

##### Exclusion criteria

1. Discharge from hospital within 24 h of admission.
2. Patients from the suburbs of each district/county in the third circle of Chengdu and refuse to go to the hospital for follow-up.

#### Sample size calculation

The sample size for this study is 120 -160 within a period of 1 year, based on the inclusion rate of HFMD hospitalized patients in previous longitudinal studies [18], the number of hospitalized patients with HFMD in 2020, and a 20 percent loss to follow-up. In addition, according to previous studies, the multi-level model could guarantee relatively accurate parameter estimations when the high level sample is not less than 50 [19, 20].

### Study procedure

**Fig. 1:**
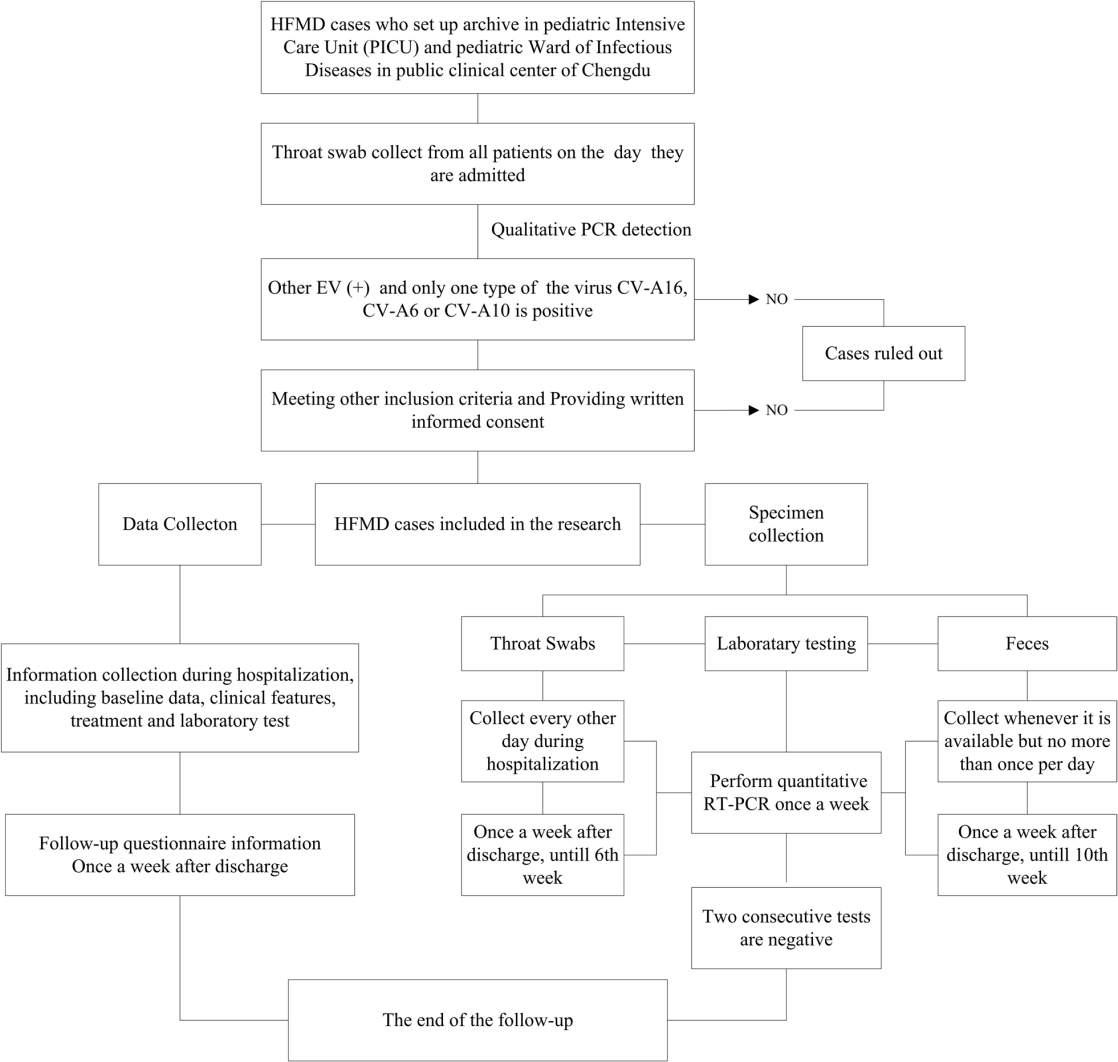
The follow chart of this study

Study subjects enrolled for this study need to conduct the procedures as below. Figure 1 provides an overview of the study procedure.

#### Enrollment of subjects

The throat swabs of all HFMD cases will be collected on the day they are admitted to clinical public center of Chengdu to conduct qualitative PCR detection to determine virus types. Every patients singly induced by CV-A6, CV-A10 or CV-A16 will be approached after laboratory determined as soon as possible. All patients providing with written informed consent from their parent or legal guardian and/or themselves will be included in the study. We will uniformly code all patients who voluntarily participate in this project.

#### Clinical data collection

A structured hospitalization questionnaire including four parts will be used to collect detailed clinical data, which spans the length of a typical course of illness. In the first part, baseline data including demographics, medical history, birth history, symptoms and signs at the onset, treatment before admission and history of EV-A71 vaccination will be collected through interview of the patients and their parents. In the second part, clinical data including detailed clinical features and complications will be collected through ward rounds, and updated every day during hospitalization. The last two parts are clinical laboratory tests and treatments, which will be exported and collected from the electronic medical record system after patients discharge from the hospital. The data collection will begin as soon as possible after the cases enter the study.

After discharge, a structured follow-up questionnaire is used to investigate the patient's health status since the last follow-up, including the occurrence of suspected HFMD symptoms, medical history, diagnosis results, and the occurrence of other HFMD cases in the same family.

#### Clinical specimen collection

During hospitalization, throat swabs will be collected once every other day, while feces will be collected whenever it is available but no more than once per day as long as the volume meets the requirement. After discharge, we will conduct a home follow-up to collect throat swabs and feces once a week until two consecutive test results are negative, or reach the end of follow-up time (6th week after admission for throat swabs and 10^th^ week for feces). All specimens will be collected according to the operating standards of the National Center for Disease Control and Prevention. All throat swabs and stool will be coded and stored in the 4 °C refrigerator temporarily (no more than 24 h) before transferred to − 80 °C freezer.

### Laboratory test

#### Viral RNA extraction

The preprocessing of all the clinical specimens and extraction of viral nucleic acid will perform in Level 2 Biosafety laboratory (BSL-2). For throat swabs samples, Mix the virus sampling tube on a vortex shaker for at least 10 s to wash off the adhering virus or virus-containing cells, the mixture will be taken directly for RNA extraction. For stool samples, a stool suspension will be made by adding a bean size feces in to 700μL normal PBS buffer and mechanically oscillate for 30 s to make the sample fully mixed. The suspension will be then centrifuged at 12,000 rmp for 5 min, and the upper suspension will be used for RNA extraction. ABT Viral RNA extraction Kit (ABT, Beijing, China) will be used for RNA extraction in the development of the assay and RNA extraction from clinical specimens according to the manufacturer’s instructions. The extracted viral RNAs will be immediately used for real-time fluorescence quantitative PCR detection. If not, the RNAs will be eluted with 40 µl of diethyl pyro carbonate-treated water and kept at − 80 °C until further use for real-time RT-PCR.

#### Real-time fluorescence quantitative PCR

The viral load of CV-A6, CV-A10 and CV-A16 will be detected by real-time fluorescence quantitative reverse transcription polymerase chain reaction (RT-PCR). The RT-PCR kit is produced by ABT Biotechnology Company (Beijing, China), the experimental operation will be carried out according to the manufacturer’s instructions. The instrument is BIO-RAD CFX96 real-time fluorescence quantitative PCR instrument.

### Outcome measures

#### Primary outcome

The primary outcome of the study is the dynamic changes of viral load and the duration of viral shedding in HFMD patients under the influence of related factors. To obtain this result, we will first identify the positive rate and virus load of the specific serotype enterovirus at each collection time points and the time of turning negative of HFMD patients. Then, we will use Bayesian multilevel model to fit the influence of nested fixed and random factors on the variation of viral load.

#### Secondary outcomes

The secondary endpoints include number of days spent in hospital, the proportion of clinical manifestation and their duration, as well as the epidemic features of hospitalized HFMD patients induced by CV-A6, CV-A10 or CV-A16.

### Statistical analysis

We will use epidata3.1 to enter the data and R4.0.1 to conduct statistical analysis. We will follow the “Reporting of studies Conducted using ‘Observational Routinely Collected Health Data’ Statement” [21]. Our primary analysis will be the dynamic changes of viral load and the duration of viral shedding in HFMD patients, while clinical features and epidemic features of hospitalized HFMD cases will be secondary analysis.

#### Baseline characteristics

For continuous variables, we will summarize them using means ± standard deviation and the comparison between the two groups will use independent sample t-test if the data conform the normal distribution. If not, we will describe interquartile ranges, and nonparametric test will be used to compare the two groups. For categorical variables, we will report data with proportion (%) and compare groups through standard Pearson’s, or Fisher’s exact test if either group contains less than 10 patients. or Fisher’s exact test if either group contains less than 10 patients. The level of significance will be set at p < 0.05.

#### Bayesian multilevel model

Bayesian multilevel model will be used to reflect the dynamic changes of viral load and the duration of viral shedding in HFMD patients under the influence of related factors. Because of the structural nesting and unbalanced characteristics of the research data, we will use Bayesian multilevel model to fit the influence of nested fixed and random factors on the variation of viral load.

### Data management and publication

Initial study data collection is expected to be completed in late 2022. De-identified data is expected to be available a month after that. All data will be collected through paper questionnaires and entered manually by the research team. The database will be stored in an encrypted manner with Epidata3.1 limited to authorized personnel only, and the paper questionnaire will be sealed in the project office. Scientific publications or reports will not identify individual participants.

Results of the study will be submitted for publication in international peer reviewed biomedical journals. Position of authorship will be determined according to international standards for authorship in peer-reviewed journals.

### Study status

All implementation site and laboratory preparations have been completed. Pre-investigation is currently conducting and will be finished on January 31, 2022, and the samples collected in pre-investigation will be used to laboratory operation process training. Formal investigation and participant recruitment will be carried out from February 1 to December 31, 2022.

## Discussion

This protocol describes a longitudinal study which aims to assess the law of the viral shedding in HFMD patients. Until now, there were few quantitative studies on the changes of viral load and the law of the viral shedding in difference specimens of HFMD patients induced by difference viral types has not yet been clarified. As one of the few studies with a long-term follow-up design to quantitative determinate the viral load in deference samples of HFMD patients, the results of our research will fill up the gaps in research on the dynamic changes of viral load in patients with HFMD.

The dynamic change process of viral load in the body is affected by many factors including serotypes, specimen types, severity and treatment, thereby affecting the duration of viral shedding [22, 23]. In this study, we will obtain comprehensive data including detailed clinical characteristics, laboratory test results and treatments through prospectively collect and update. And we will quantify the viral load in throat swabs and feces from each patient during the entire detox period through follow-up. The results of our research can further clarify the evolvement rules which reflect the dynamic changes of viral load and the duration of viral shedding in HFMD patients under the influence of related factors.

Moreover, the site of the project is the designated hospital for inpatients with HFMD in Chengdu, and almost all inpatients will be referred to this hospital for treatment. This study will be based on a well-characterized sample of subjects with HFMD, who are assessed for a broad number of predictor variables at baseline including clinical features, laboratory test and treatment. And details of symptoms and signs which may influence outcome will be assessed over the illness course.

There are also undeniable limitations to this study. The major limitation might be the loss of follow-up, just like any other follow up study cannot avoid. Especially because the Clinical Public Health Center of Chengdu is a designated treatment and isolation hospital for COVID-19 patients in Sichuan Province, many patients may be unwilling to return to the hospital for return visits. From this perspective, we will conduct home follow-up visits by trained project members to complete sample collection. In addition, during the study recruitment process, the investigator will explain that enrollment in the cohort requires continued follow-up. Subjects with a strong willingness to participate will be included, which may avoid loss to follow-up. Appropriate method will be used to deal with the lost data and assess its impact on the results.

On the other hand, the clinical public health center of Chengdu is a regional referral hospital for HFMD management, so some patients might be already on the 2–3th days after the onset of illness when admitted to the hospital. Therefore, for these patients enrolled in the study, the clinical specimens on the first day of illness cannot be obtained. This situation does not influence the observation of virus excretion duration, but it will have a certain impact on the early dynamic changes of viral load. In order to minimize the impact, we will give priority to patients admitted to the hospital on the day of onset during recruitments.

## Data Availability

The anonymized datasets generated during the current study will be made available from the corresponding author on reasonable request.

## Abbreviations

HFMD: Hand, foot and mouth disease
EV-A71: Enterovirus A71
CV-A16: Coxsackievirus A16
CV-A6: Coxsackievirus A6
CV-A10: Coxsackievirus A10
STROBE: Strengthening the Reporting of Observational Studies in Epidemiology
BSL-2: Level 2 Biosafety Laboratory
RT-PCR: Reverse transcription polymerase chain reaction
